# The use of leukocytes’ secretome to individually target biological therapy in autoimmune arthritis: a case report

**DOI:** 10.1186/s40169-019-0236-7

**Published:** 2019-06-05

**Authors:** Patrice E. Poubelle, Nathalie Pagé, Marie-Pier Longchamps, Natalia Sampaio Moura, David B. Beck, Ivona Aksentijevich, Philippe A. Tessier, Martin Pelletier

**Affiliations:** 1 Axe de recherche sur les maladies infectieuses et immunitaires, Centre de recherche du CHU de Québec-Université Laval, Room T1-49, 2705 Boul. Laurier, Québec, QC G1V 4G2 Canada; 2 Département de médecine, Faculté de Médecine, Université Laval, Québec, QC G1V 0A6 Canada; 3 Metabolic, Cardiovascular and Inflammatory Disease Genomics Branch, National Human Genome Research Institute, National Institutes of Health, 10 Center Drive, Building 10, Room B3-4129, Bethesda, MD 20892-1852 USA; 4 Département de microbiologie-infectiologie et d’immunologie, Faculté de Médecine, Université Laval, Québec, QC G1V 0A6 Canada; 5 Metabolic, Cardiovascular and Inflammatory Disease Genomics Branch, National Human Genome Research Institute, National Institutes of Health, 10 Center Drive, Building 10, Room B2-5235, Bethesda, MD 20892-1852 USA

**Keywords:** Biologic agents, Interleukin-6, Personalized medicine, Secretome, Leukocytes, Arthritis, TRAF3IP2, TRAF6

## Abstract

**Background:**

Biological agents have allowed remarkable improvement in controlling autoimmune arthropathies, although none of the numerous biologics readily available represent a universal treatment standard. Moreover, classical and genetic predictors are currently unsatisfactory to predict individual response to a biologic, and the best treatment selection is still based on a trial-and-error approach. Here, we report a clinical case demonstrating the usefulness of examining the leukocytes’ secretome of patients. We set up and standardized a protocol that examines a patient’s immune responses to establish the secretome of the blood mononuclear leukocytes and personalize the biotherapy.

**Case presentation:**

A 24-year-old woman was diagnosed with active early rheumatoid arthritis. The initial treatment regimen (prednisone, methotrexate, hydroxychloroquine, naproxen) was inefficient, as well as the anti-TNF adalimumab. The diagnosis was revised as possible rheumatoid arthritis-like psoriatic arthritis and adalimumab was replaced by abatacept (IgG1 Fc-CTLA-4) to no avail. Five years later, abatacept was replaced by the anti-IL-12/IL-23 ustekinumab with no objective control over the symptoms. The patient was thus enrolled in a prospective study based on the quantification of cytokines secreted by peripheral blood leukocytes stimulated with well-known immune activators of pattern recognition receptors and cytokine signalling. The results of this study revealed that plasma concentrations of cytokines were similar between the patient and healthy donors. In comparison to leukocytes from healthy donors, the patient’s secretome showed a unique overproduction of IL-6. The anti-IL-6 receptor tocilizumab was, therefore, administered with a rapid improvement of her active psoriatic arthritis that remained dependent on low prednisone dosage. Clinical parameters progressively returned to normal levels and her quality of life was greatly improved, despite the major delay to begin the present personalized treatment.

**Conclusions:**

An efficient way to effectively treat patients with complex autoimmune arthropathies, and avoid irreversible disability, is to know their leukocytes’ secretome to identify abnormally secreted cytokines and personalize their biotherapy, as exemplified by this case report.

## Background

Autoimmune arthropathies are treated with biologics that allowed remarkable advancement in the control of disease alteration [1]. However, some patients do not respond to the first biologic, or even to the others successively given with or without methotrexate and studies to identify predictors of response to biologic therapy are still limited [2]. The classical predictors such as the presence of auto-antibodies [the rheumatoid factor (RF) and the anti-cyclic citrullinated peptide (anti-CCP)], genotypes encoding the shared epitope *HLA*-*DRB1* gene, smoking and/or periodontitis are largely insufficient to foresee the patient response to a biologic [3]. Genetic predictors represent an ongoing field of research and bear the potential to contribute to the development of a precision medicine approach in the management of autoimmune arthropathies [4, 5]. Nonetheless, the identification of genetic markers of disease outcome and response to treatment is still at its infancy and has been somewhat disappointing so far [6].

The most fruitful findings to treat autoimmune arthropathies remain the characterization of immune mediators involved in the disease. This is greatly supported by the numerous biologics readily available to treat most of the autoimmune diseases, including biotherapies targeting specific immune cells, such as the cytotoxic T-lymphocyte-associated protein-4 (CTLA-4, also known as CD152) or the B-lymphocyte antigen CD20, or secreted mediators like the pro-inflammatory cytokines tumor necrosis factor (TNF), interleukin (IL)-1, IL-6, IL-12/IL-23 and IL-17 [7]. Although a range of treatment options can be addressed with biologics, none of them are universally effective and the best treatment selection is still based on a trial-and-error approach, where the most suitable one is determined when a drug reduces disease activity or remission is identified [8]. Considering the severity of these life-threatening diseases and the high cost of biologics, the best treatment option should target, from the start, the patients’ own pattern of cytokines [9].

Here, we report a clinical case demonstrating the usefulness of examining the leukocytes’ secretome of patients. We set up and standardized a protocol that investigates the immune responses of the patients to establish the secretome of their blood mononuclear leukocytes. The results were used to personalize the biotherapy of a patient suffering from an autoimmune arthropathy, providing insights on how to tailor the best treatment option and therefore avoid definitive disability and loss of quality of life.

## Case presentation

A 24-year-old woman was examined for the first time 3 months after the onset of symmetrical polyarthritis with major synovitis of 2nd, 3rd, 4th metacarpophalangeal joints of both hands, wrists, elbows, knees, ankles, forefeet, without any spinal signs. The disease activity score of 28 joints (DAS28) and DAS28 using the C-reactive protein (DAS28-CRP) were 8.09 and 7.75, respectively. Increased ferritin and thrombocytosis in the absence of detectable levels of RF, anti-CCP and antinuclear antibody (ANA) were also noticeable. Her liver function tests and lipid panel were normal and no bone erosion was visible by X-rays. She was diagnosed with active early rheumatoid arthritis (RA) (Table 1).

**Table 1:** Patient information and diagnostics summary

Time since onset of disease	3 months	1 year	2 years	5 years	10 years	11 years
Clinical parameters						
DAS28	8.09	8.37	7.94	6.87	7.61	3.76
DAS28-CRP	7.75	7.98	7.55	6.63	7.44	4.34
Bone erosions at X-rays	None	None	Yes	Yes	Yes	Yes
Biological parameters						
ESR (mm, first hour; Nl < 20)	63	68	37	8	24	5
CRP (mg/L; Nl < 10)	56	85	25	1	18	7
Ferritin (μg/mL; Nl < 200)	347	314	202	141	165	Not evaluated
Platelet count (Nl < 400 billion/L)	469	390	348	225	247	263
RF	Undetectable	Undetectable	Undetectable	Undetectable	Undetectable	Undetectable
Anti-CCP	Undetectable	Undetectable	Undetectable	Undetectable	Undetectable	Undetectable
ANA	Undetectable	Undetectable	Undetectable	Undetectable	Undetectable	Undetectable
Synovial fluid analysis		8.93 billion WBC/L	21.15 billion WBC/L	120.8 billion WBC/L		
		99% neutrophils	99% neutrophils	83% neutrophils		
Diagnosis	Active early RA	Active early RA	Possible RA-like PsoA	RA-like PsoA with bone erosions	RA-like PsoA with bone erosions	RA-like PsoA with bone erosions
Treatment prescribed	Prednisone Methotrexate Hydroxychloroquine Naproxen	Initial treatment + adalimumab (anti-TNF)	Stop adalimumab; Initial treatment + abatacept (IgG1 Fc-CTLA-4)	Initial treatment + abatacept (IgG1 Fc-CTLA-4)	Stop abatacept; Initial treatment + ustekinumab (anti-IL-12/IL-23)	Initial treatment + tocilizumab
Notes	HLA-B27 absent Treatment at visit: diclofenac	Sterile synovial fluid was removed by joint aspiration	Sterile synovial fluid was removed by joint aspiration Mother and maternal grandmother have psoriasis An aunt and her son have inflammatory spondylarthritis	Appearance of psoriasis skin lesions Sterile synovial fluid was removed by joint aspiration Ustekinimab (anti-IL-12/IL-23) and secukinimab (anti-IL-17) were not authorized at the time	No improvement after 6 months treatment with ustekinumab Stop ustekinumab Enrollement in study to quantify cytokines in plasma and secreted by activated PBMCs Tocilizumab (anti-IL-6) begun	

*ANA* antinuclear antibody, *anti-CCP* anti-cyclic citrullinated peptide, *CRP* C-reactive protein, *DAS28* disease activity score of 28 joints, *ESR* erythrocyte sedimentation rate, *Nl* normal level, *PsoA* psoriatic arthritis, *RA* rheumatoid arthritis, *RF* rheumatoid factor, *WBC* white blood cells

Initial treatments with prednisone, methotrexate, hydroxychloroquine and naproxen were without efficacy. The anti-TNF adalimumab was added to the treatment regimen for 2 years. After only mild improvement, she experienced a progressive flare-up of polyarthritis and a loss of treatment efficacy. Two years after the onset of the disease, wrist and tarsal (right and left) demineralization, as well as bone erosions of ulnar styloids (right and left), appeared. Erythrocyte sedimentation rate (ESR), CRP and ferritin were persistently increased while RF and anti-CCP remained undetectable. The diagnosis was revised as possible RA-like psoriatic arthritis (PsoA), especially as her mother has skin psoriasis. Bone lesions were increased rapidly, in particular at both wrists. Adalimumab was replaced by abatacept (IgG1 Fc-CTLA-4) with a mild effect on arthritis. Five years after disease onset, psoriatic skin lesions appeared, and diagnosis of cutaneous psoriasis was confirmed by a dermatologist. The final diagnosis was aggressive RA-like PsoA with bone erosions, without RF and anti-CCP. Abatacept was replaced by the anti-interleukin (IL)-12/IL-23 ustekinumab with an increase of prednisone dosage. A mild relieve of polysynovitis was noted, which was dependent on prednisone. Reduction of prednisone led to a major flare-up of polysynovitis associated with asthenia, and after 3 months of ustekinumab administration, no objective effect on the patient’s symptoms was noted.

The patient was enrolled in a prospective study based on the quantification of cytokines secreted by peripheral blood leukocytes. Blood (50 mL) of the patient as well as of healthy donors was drawn after informed consent was obtained. Plasma was collected following centrifugation (400×*g* for 10 min) of anti-coagulated blood and stored at − 80 °C for further protein quantification. Peripheral blood mononuclear cells (PBMCs) were obtained following centrifugation (600×*g* for 20 min) of the cellular fraction of blood over density gradient medium (Lymphocyte separation medium, density 1.077–1.080 g/mL; Wisent Bioproducts Inc., St-Bruno, Québec, Canada). Density gradient-purified PBMCs were stimulated with well-known immune activators of pattern recognition receptors and cytokine signalling for 24 h at 1 × 10^6^/mL in RPMI 1640 (Wisent Bioproducts Inc., St-Bruno, Québec, Canada) supplemented with 10% fetal bovine serum (VWR Life Science Seradigm, Mississauga, Ontario, Canada) and 1% primocin (InvivoGen, San Diego, California, USA) in the absence (control) or presence of plate-bound mouse anti-human CD3 (1 μg/mL, clone OKT3) + anti-human CD28 (10 μg/mL, clone 9.3), lipopolysaccharides (LPS—100 ng/mL; 45 nM, InvivoGen, San Diego, California, USA) + adenosine triphosphate (ATP—1 mM; added for the last 30 min, Sigma-Aldrich Canada Co., Oakville, Ontario, Canada), L18-muramyl dipeptide (L18-MDP—1 μg/mL; 1.32 μM, InvivoGen, San Diego, California, USA), Poly(deoxyadenylate–thymidylate) [Poly(dA:dT)—1 μg/mL; 1.57 μM, InvivoGen, San Diego, California, USA], anisomycin (20 μM, Millipore (Canada) Ltd, Etobicoke, Ontario, Canada) or pro-inflammatory cytokines IL-1β (100 ng/mL; 5.85 nM, PeproTech US, Rocky Hill, New Jersey, USA), TNF (100 ng/mL; 5.85 nM, STEMCELL Technologies Canada Inc., Vancouver, British Columbia, Canada), IL-6 (100 ng/mL; 3.83 nM, PeproTech US, Rocky Hill, New Jersey, USA) and IFN-γ (100 U/mL; 0.30 nM, PeproTech US, Rocky Hill, New Jersey, USA) to respectively activate T cells, the NLRP3, NOD2, AIM2 and pyrin inflammasomes as well as cytokine signalling. Following stimulation, cell supernatants were collected and stored at − 80 °C for further protein quantification. Proteins involved in inflammation (IL-1α, IL-1β, IL-6, IL-9, IL-15, IL-17A, IL-18, IL-21, IL-31, TNF, LT-α, IFN-γ), immunoregulation (IL-1RA, IL-4, IL-7, IL-10, IL-12, IL-13, IL-22, IL-23, IL-27, IFN-α), chemotaxis (CCL2/MCP-1, CCL3/MIP-1α, CCL4/MIP-1β, CCL5/RANTES, CCL11/Eotaxin, CXCL1/GROα, CXCL8/IL-8, CXCL10/IP-10, CXCL12/SDF-1α) and cellular growth (IL-2, IL-5, GM-CSF) were quantified by multiplex analyses in plasma and cell supernatants using Luminex technology according to the manufacturers’ instructions (Cytokine & Chemokine 34-Plex Human ProcartaPlex™ Panel 1A, Thermo Fisher Scientific Inc., Burlington, Ontario, Canada).

**Fig. 1:**
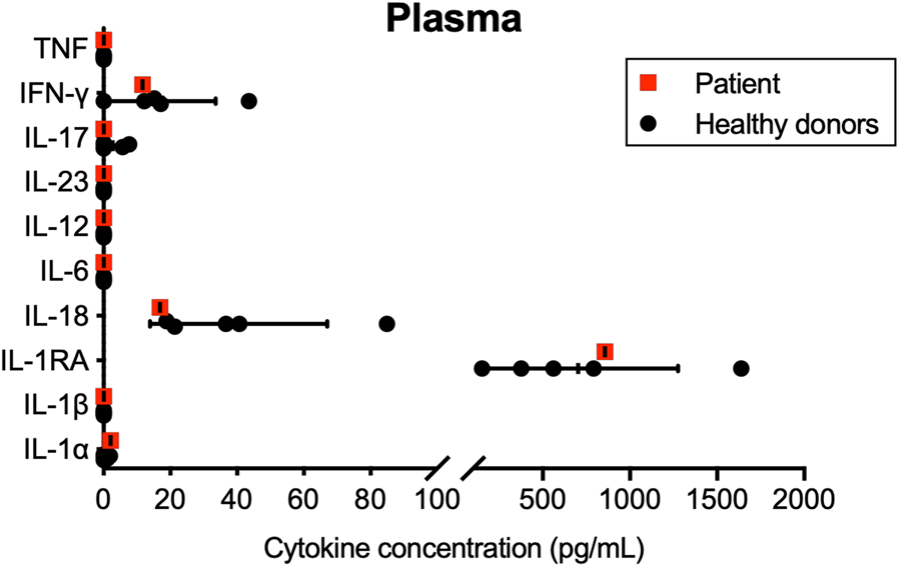
Similar levels of cytokines in the plasma of the patient and healthy donors. Concentrations of IL-1α, IL-1β, IL-1RA, IL-18, IL-6, IL-12, IL-23, IL-17, IFN-γ and TNF were determined by multiplex assays in the plasma of healthy donors (black dots) and the patient with aggressive RA-like PsoA (red square). Results are expressed as mean ± standard deviation for the healthy donors (n = 5)

**Fig. 2:**
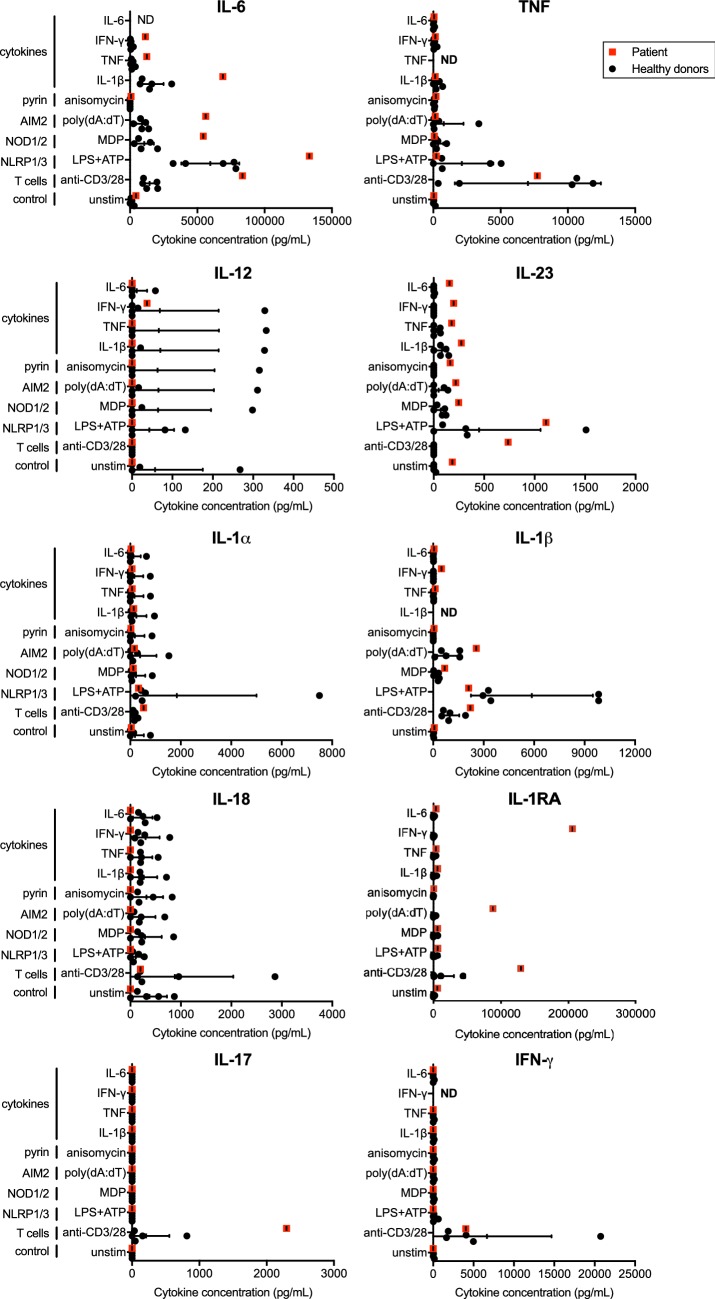
Aberrant production of IL-6 by the patient’s blood leukocytes. Density gradient-purified peripheral blood mononuclear cells were left unstimulated (unstim) or stimulated for 24 h with anti-CD3 + anti-CD28, lipopolysaccharide (LPS) + adenosine triphosphate (ATP), L18-muramyl dipeptide (L18-MDP), poly(deoxyadenylate–thymidylate) [poly(dA:dT)], anisomycin or the pro-inflammatory cytokines IL-1β, TNF, IFN-γ and IL-6 to respectively activate T cells, the NLRP3, NOD2, AIM2 and pyrin inflammasomes as well as cytokine signalling. Concentrations of IL-6, TNF, IL-12, IL-23, IL-1α, IL-1β, IL-18, IL-1RA, IL-17 and IFN-γ were determined by multiplex assays in the cells’ supernatants of healthy donors (black dots) and the patient with aggressive RA-like PsoA (red square). Results are expressed as mean ± standard deviation for the healthy donors (n = 5). ND; not determined

The results of this study revealed that plasma concentrations of cytokines were similar between the patient and healthy donors (Fig. 1 and data not shown). In comparison to leukocytes from healthy donors, the patient’s secretome showed a unique overproduction of IL-6 in response to multiple stimuli, including the inflammasome activators LPS + ATP, MDP and poly(dA:dT), as well as the pro-inflammatory cytokines IL-1β, TNF and IFN-γ, to levels (up to 133,000 pg/mL) at least twice the ones produced by healthy donors’ cells (Fig. 2). This overproduction of IL-6 occurred without substantial increase of pro-inflammatory cytokines such as TNF, IL-12 and IL-23, which correlates with the inefficacy of the anti-TNF adalimumab and the anti-IL-12/IL-23 ustekinumab treatments. No substantial differences were observed for members of the IL-1 cytokine family (IL-1α, IL-1β, IL-18), as well as IFN-γ and IL-17. Increased secretion of the IL-1 receptor antagonist (IL-1RA) was shown upon stimulation with the AIM2 inflammasome activator poly(dA:dT) as well as IFN-γ, suggesting that the patient’s leukocytes can synthesize high amounts of IL-1RA to neutralize the production of IL-1. Of note, the stimulation of the patient’s T cells using a combination of anti-CD3 and anti-CD28 also led to increased production of IL-6, as well as IL-23, IL-1RA and IL-17, but not to the levels observed for IL-6 (Fig. 2). Finally, no substantial differences were observed between the patient and the healthy donors regarding the production of IL-2, IL-4, IL-5, IL-7, IL-9, IL-10, IL-13, IL-15, IL-21, IL-22, IL-27, IL-31, LT-α, IFN-α, CCL2/MCP-1, CCL3/MIP-1α, CCL4/MIP-1β, CCL5/RANTES, CCL11/Eotaxin, CXCL1/GROα, CXCL8/IL-8, CXCL10/IP-10 and CXCL12/SDF-1α by unstimulated or stimulated PBMCs (data not shown).

Thus, considering the unique major overproduction of IL-6 by the patient’s leukocytes, the anti-IL-6 receptor tocilizumab was administered with a rapid improvement of her active PsoA that remained dependent on low prednisone dosage. DAS28 and DAS28-CRP were greatly improved at 3.76 and 4.34, respectively. ESR, CRP and ferritin were progressively normalized. Her quality of life was greatly improved with, in particular, a progressive reduction of asthenia. Have the patient been enrolled in this prospective study sooner, her refractory PsoA would have probably benefited from the personalized treatment without the current associated irreversible destructive arthritis and partial functional handicap, especially at both wrists.

## Discussion

The immunological disease continuum, that includes all autoinflammatory and autoimmune diseases, as proposed by McGonagle & McDermott [10], allows to better understand the complexity of immune factors that can be associated with the numerous immunological disorders. In this regard, the present case report can possibly be related to polygenic autoinflammatory diseases and mixed pattern diseases, both conceptual classification in which psoriatic arthritis can be found. The investigation of the patient’s secretome, as we report here, remains non-exhaustive, with a major focus on the biologics currently available to treat autoimmune arthropathies such as RA and PsoA (Table 2). This focus could easily be extended to other factors depending on new biologics, as well as other stimuli to activate relevant immune signalling as the field progresses further. Nonetheless, the targeted secretome allows deciphering whether a factor could be largely produced over others to be able to adapt more precisely the treatment. This case report exemplifies the personalized treatment option based on the patient’s medical history and the determination of the secretome of the patient’s blood leukocytes in response to immune activators that revealed a unique overproduction of the IL-6 cytokine. In fact, the overproduction of IL-6 in the present case report of a refractory PsoA was in line with reports of mediators from synovial inflammation of PsoA where anti-IL-6 biologics were effective treatment choices [11–15].

**Table 2:** Biotherapies and small molecule inhibitors against secreted mediators or immune cells and their approval for rheumatoid arthritis and psoriatic arthritis

Target	Agent	Structure	Mechanism of action	Approval for RA		Approval for PsoA	
				FDA	Health Canada	FDA	Health Canada
TNF	Etanercept	Fusion protein Fc IgG1-TNFR2	Decoy receptor	Nov 1998	Dec 2000	Jan 2002	Mar 2004
	Infliximab	Chimeric mAb to TNF	Antagonist	Nov 1999	Sep 2001	May 2005	Jun 2006
	Adalimumab	Humanized mAb to TNF		Jan 2003	Sep 2004	Oct 2005	Jun 2006
	Golimumab	Human mAb to TNF		Apr 2009	Apr 2009	Oct 2017	Apr 2009
	Certolizumab pegol	Pegylated Fab’ fragment of humanized mAb to TNF		May 2009	Sep 2009	Sep 2013	Jan 2014
IL-1	Anakinra	Recombinant human IL-1 receptor	Receptor antagonist	Nov 2001	Jun 2002	–	–
	Canakinumab	Human mAb to IL-1β	Antagonist	–	–	–	–
	Rilonacept	Fusion protein Fc IgG1 linked to ligand-binding domains of IL-1R1 and IL-1RAcP					
CTLA-4	Abatacept	Fusion protein Fc IgG1-CTLA-4	Binds CD80 and CD86 on APCs; prevents co-stimulation of T cells	Dec 2005	Jun 2006	Jul 2017	Mar 2018
CD20	Rituximab	Chimeric mAb to CD20	Binds CD20 on B cells; triggers cell death	Feb 2006	Jun 2006	–	–
	Ocrelizumab	Humanized mAb to CD20		–	–	–	–
	Ofatunumab	Human mAb to CD20		–	–	–	–
	Obinutuzumab	Humanized mAb to CD20 with a glycoengineered Fc		–	–	–	–
IL-6	Tocilizumab	Humanized mAb to IL-6 receptor	Receptor antagonist	Jan 2010	May 2010	–	–
	Sarilumab	Human mAb to IL-6 receptor		May 2017	Feb 2017	–	–
IL-12, IL-23	Ustekinumab	Human mAb to IL-12 and IL-23 p40 subunit	Antagonist	–	–	Sep 2013	Jan 2014
IL-17	Secukinumab	Human mAb to IL-17A	Antagonist	–	–	Jan 2016	Apr 2016
	Ixekizumab	Human mAb to IL-17A		–	–	Dec 2017	Jun 2018
	Brodalumab	Human mAb to IL-17 receptor A	Receptor antagonist	–	–	–	–
Janus kinase 1–3	Tofacitinib	Small molecule	Intracellular inhibitor; blocks activity of cytokines (IL-2, -4, -6, -7, -9, -10, -12, -15, -21, -23, IFN-α, -β, -γ)	Nov 2012	Apr 2014	Dec 2017	Oct 2018

**Fig. 3:**
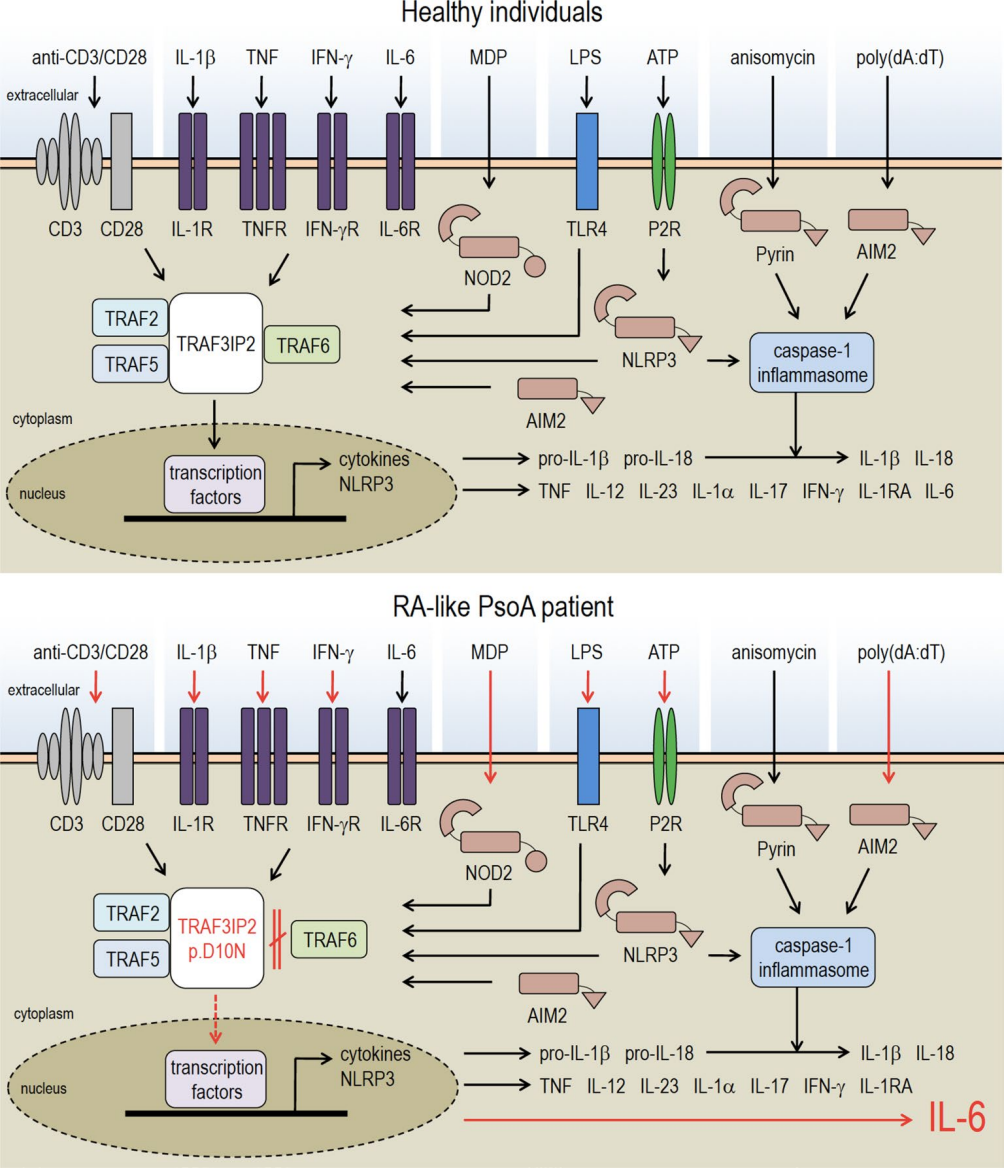
IL-6 production by blood leukocytes is altered in the RA-like PsoA patient in response to immune activators. In healthy individuals (top panel), the stimulation of peripheral blood mononuclear cells with immune activators leads to the activation of intracellular multiprotein oligomers (e.g. inflammasomes) and specific receptors that activate signalling pathways, including TRAF proteins, translocation of transcription factors and subsequent cytokine response. In the patient with aggressive RA-like PsoA (bottom panel), the activation of blood cells with anti-CD3 + anti-CD28, pro-inflammatory cytokines IL-1β, TNF and IFN-γ, muramyl dipeptide (MDP), lipopolysaccharide + adenosine triphosphate (LPS + ATP) and poly(deoxyadenylate–thymidylate) [poly(dA:dT)] led to a unique overproduction of IL-6 (red arrows). As the patient is a heterozygous carrier for a single nucleotide polymorphism p.Asp10Asn (p.D10N) in *TRAF3IP2*, this susceptibility allele causes altered TRAF6 binding (double red lines with an oblique stroke). The inhibition of the TRAF6 pathway could upregulate the TRAF2/5 pathway, leading to an enhanced immune response [21, 22] (dashed red arrow)

Another consideration is at the level of a unique overproduction of IL-6 in the present case of refractory PsoA, which could indicate a predominant autoinflammatory pattern over the autoimmune part of PsoA. In this regard, genomic DNA samples were isolated from the patient’s peripheral blood, and whole exome sequencing and data analysis were performed on the proband as previously described [16]. Publicly-available databases (ExAC, 1000 genomes, dbSNP, NHLBI GO Exome Sequencing Project and ClinSeq), as well as an in-house exome database, were used to filter for rare candidate variants. We did not identify any pathogenic mutations in known disease-causing genes. We analyzed the sequencing data for novel causes of immune dysregulation accounting for different modes of inheritance. Ideally, whole exome sequencing should be performed in trios to help with filtering a long list of candidate variants that are typically found when doing exome sequencing in singleton cases. Nonetheless, we did not identify any strong candidate variants to explain the phenotype. We also performed Sanger sequencing to analyze specific non-coding polymorphisms in the *IL6* promoter and 5′UTR that could potentially account for some of the patient’s phenotype since the clinical spectrum of PsoA, more precisely the active peripheral arthritis, has been linked to IL6 (-174G/C) polymorphism [17]. Coding regions as well as flanking 5′ untranslated regions of the *IL6* gene (RefSeq: NM_000600.5) were amplified by AmpliTaq Gold Fast PCR Master Mix (Thermo Fisher Scientific Inc., Waltham, Massachusetts, USA) and sequenced on SeqStudio Genetic Analyzer (Applied Biosystems Inc., Foster City, California, USA). We found that the patient had reference alleles at -597G, -572G, -473A8/T12 and -174G [18]. We finally analyzed the data for common low-penetrance coding variants associated with psoriatic or rheumatoid arthritis. We found that the patient is a heterozygous carrier for the single nucleotide polymorphism (SNP) in *TRAF3IP2* rs33980500 (NM_001164281.2:c.28G>A; p.Asp10Asn; NM_147200.2:c.55G>A, NP_671733.2;p.Asp19Asn), which was identified by two genome-wide association studies (GWAS). The susceptibility allele rs33980500 has been shown to cause altered TRAF6 binding and thereby affect multiple immune pathways [19, 20]. Because TRAF3IP2 binding motifs have been associated with multiple TRAF proteins, the inhibition of the TRAF6 pathway could upregulate the TRAF2/5 pathway, leading to an enhanced immune response. Interestingly, studies showed that, in the TRAF6-independent pathway, TRAF2 and TRAF5 transduce the IL-17 signals to stabilize mRNA transcripts of chemokines and cytokines, such as CXCL1/GROα and IL-6 [21, 22]. Although these results did not identify a high penetrance genetic cause, they did identify a risk allele in *TRAF3IP2*, which in combination with additional environmental and genetic factors may help to explain this patient’s phenotype and suggest a further link between the PsoA, IL-6 production and TRAF3IP2/TRAFs signalling (Fig. 3).

While we examined the secretome of blood cells, one limitation of this study is that many other cell types in autoimmune arthritis are known to secrete cytokines, such as synoviocytes, fibroblasts, chondrocytes, osteoblasts/osteoclasts and epithelial cells (the latter ones in psoriatic arthritis for example), and that these cells, through their cytokine secretion, could contribute to the local pathology [23–26]. The cytokine pattern could evidently be different from the ones produced by the blood cells. However, the use of these cells often requires invasive techniques to obtain them and their purification, for subsequent activation, is more labour-intensive than blood cells [27]. It is also useful to stress that the presence of a SNP in *TRAF3IP2* rs33980500, as shown in this case report, should be present in all cell types using this signalling pathway [28, 29].

## Conclusion

As a corollary and conclusion, an efficient way to effectively treat patients with complex autoimmune arthropathies, and to avoid irreversible disability, is to determine their blood leukocytes’ secretome to identify abnormally secreted cytokines to personalize their biotherapy. Another concluding detail should also be noted as the finding of an abnormal patients’ secretome can justify pursuing a causal investigation such as a genetic evaluation, as exemplified by this case report.

## Abbreviations

ANA: antinuclear antibody
Anti-CCP: anti-cyclic citrullinated peptide
DAS28: disease activity score of 28 joints
DAS28-CRP: DAS28 using the C-reactive protein
GWAS: genome-wide association study
IL: interleukin
PsoA: psoriatic arthritis
RA: rheumatoid arthritis
RF: rheumatoid factor
SNP: single nucleotide polymorphism
TNF: tumor necrosis factor
